# Successful transcatheter arterial embolization for massive hemorrhage from acquired uterine arteriovenous malformation which occurred as a complication of hysterectomy: A case report

**DOI:** 10.1097/MD.0000000000024052

**Published:** 2021-01-15

**Authors:** Chang Hoon Oh, Yook Kim, Bum Sang Cho, Kyung Sik Yi

**Affiliations:** a Department of Radiology; b Department of Neurosurgery, Chungbuk National University Hospital, Cheongju, Republic of Korea.

**Keywords:** embolization, hysterectomy, uterine arteriovenous malformation

## Abstract

**Rationale:**

Uterine arteriovenous malformation (UVM), which can be congenital or acquired, is a relatively rare disorder that can cause life-threatening hemorrhage. Acquired UVM occurs predominantly after previous uterine procedures; rarely, it may occur after a hysterectomy. Although the best treatment option for UVM remains controversial, transcatheter arterial embolization (TAE) has recently been introduced as a safe and effective treatment.

**Patient concerns:**

A 34-year-old woman who underwent hysterectomy for uncontrolled postpartum bleeding continued to have hemoperitoneum.

**Diagnosis:**

Two days after surgery, massive hemoperitoneum was identified on computed tomography scan, and acquired UVM was diagnosed by angiography.

**Interventions:**

The patient was successfully treated using TAE with an n-Butyl cyanoacrylate.

**Outcomes:**

After embolization, hemodynamic stability was achieved. A day after embolization, hemoglobin was 10.2 g/dL, and the patient was discharged from the hospital 4 days thereafter.

**Lessons:**

Although the overall incidence of acquired UVM after hysterectomy is low, bleeding from acquired UVM should be considered as one of the differential diagnoses in the immediate postpartum period, especially when the clinical symptoms do not correlate with the amount of blood loss. A high index of suspicion, prompt diagnosis and intervention, and a multidisciplinary approach in the management were the elements of a successful outcome in this case.

## 1 Introduction

Uterine arteriovenous malformation (UVM), which can be congenital or acquired, is a rare cause of gynecological bleeding. In 1% to 2% of cases, massive bleeding or intraperitoneal hemorrhage can occur, and this can be life-threatening.^[1–3]^ The preferred noninvasive method for diagnosing UVM is by color Doppler ultrasound. Computed tomography (CT) scan and magnetic resonance imaging (MRI) may be used to determine its size, extent and vascularity, and the involvement of adjacent organs.^[1,2]^ Traditionally, UVM has been treated by surgical ligation. However, more recently, treatment has been successfully accomplished through transcatheter arterial embolization (TAE) of the uterine artery. TAE has emerged as a highly effective percutaneous technique for controlling genital bleeding in a variety of obstetric and gynecologic disorders.^[3,5]^ Angiography enables both the precise identification and localization of bleeding vessels, and the subsequent embolization as a therapeutic option with minima invasion; therefore, trauma to the patient and the volume of damaged tissue are minimized. The major cause of acquired UVM is usually traumatic, which may be by prior dilatation and curettage, direct uterine trauma, or rarely, after hysterectomy.^[1,2]^ There are very few reported cases of UVM after hysterectomy.^[6,7]^ A review of the literature identified 13 cases, the largest of which was a 1970 case series that included 7 patients.^[1,3–7]^ Herein we report a case of massive hemoperitoneum from the acquired UVM occurring immediately after hysterectomy for uncontrolled postpartum hemorrhage that was successfully treated using TAE with *n*-Butyl cyanoacrylate (NBCA). The study design was approved by the Chungbuk National University Hospital Institutional Review Board, and the written informed consent was obtained from the patient for publication of this case report and accompanying image.

## 2 Case presentation

A 34-year-old woman (gravida 2, para 2) was referred to our hospital on account of persistent vaginal bleeding after a cesarean section she had undergone on the same day at a local obstetrics and gynecologic clinic. Her past medical history and family history were not significant. Her postpartum hemoglobin was 8.3 g/dL, which necessitated an emergency packed red blood cell transfusion. She had a blood pressure of 100/59 mmHg and a heart rate of 142/min. Angiography was performed as a primary diagnostic and therapeutic procedure. Contrary to expectations, both uterine arteries were not hypertrophied despite the recent postpartum state, and there was no evidence of vascular injuries such as active bleeding, pseudoaneurysm, or transection of blood vessel on both internal iliac artery angiographies (Fig. 1A and B ), and pelvic angiography. Considering the continuous vaginal bleeding and her unstable hemodynamic status, an emergency total abdominal hysterectomy with bilateral salpingectomy was performed right after the initial angiography, and vaginal laceration was confirmed during surgery. Pathological examination showed no abnormalities of the uterus, cervix, or fallopian tubes. Two days after the surgery, 2000 mL of blood was present in a drainage tube that had been inserted intraoperatively, and hemoglobin level declined from 8.3 to 5.2 g/dL. A dynamic CT scan performed revealed a tortuous, hypertrophied vascular structure at the right lateral aspect of the pelvic cavity (Fig. 2A) along with a large amount of hemoperitoneum (Fig. 2B). A repeat angiography of the right internal iliac artery demonstrated a complex tangling of vessels supplied by an enlarged feeding artery, and this was not seen on the first angiography (Fig. 3A). Moreover, superselective uterine artery angiography showed early venous drainage into hypertrophied veins, and stasis of contrast medium in the abnormal vascular structure, which indicated UVM (Fig. 3B). Therefore, embolization of the right uterine artery was performed using NBCA, and a post-embolization angiogram revealed successful hemostasis with disappearance of the UVM (Fig. 3C). After embolization, hemodynamic stability was achieved. A day after the embolization, hemoglobin was 10.2 g/dL, and the patient was discharged from the hospital 4 days thereafter.

**Figure 1:**
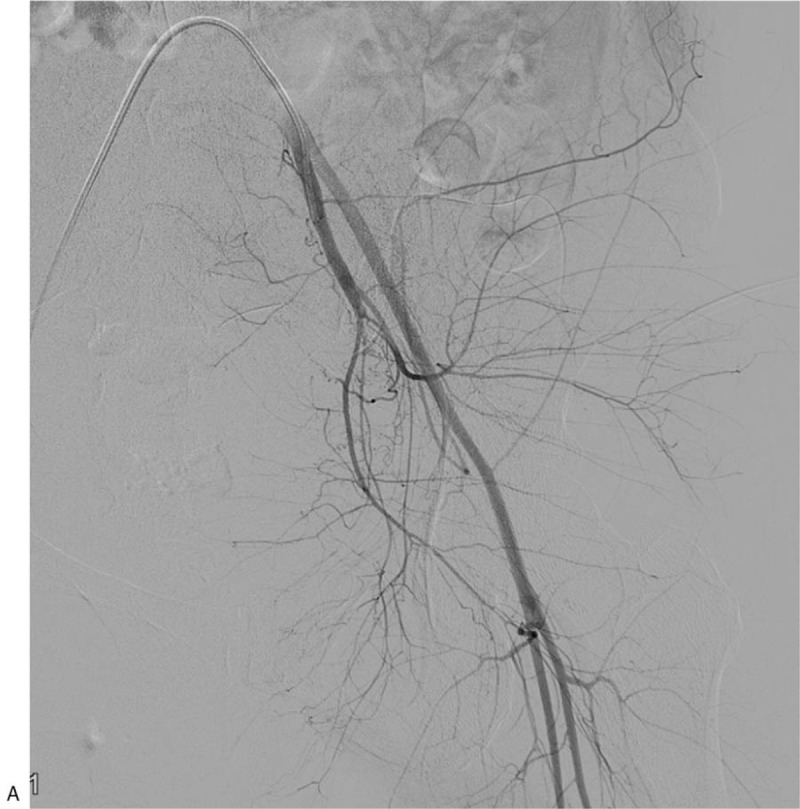
A 34-year-old woman (gravida 2, para 2) transferred to our hospital for persistent vaginal bleeding after cesarean section. (A and B) Both uterine arteries are not hypertrophied despite recent postpartum state, and there is no evidence of vascular injuries such as active bleeding, pseudoaneurysm, or transection of vessel on the angiographies of both internal iliac arteries.

**Figure 1 (Continued):**
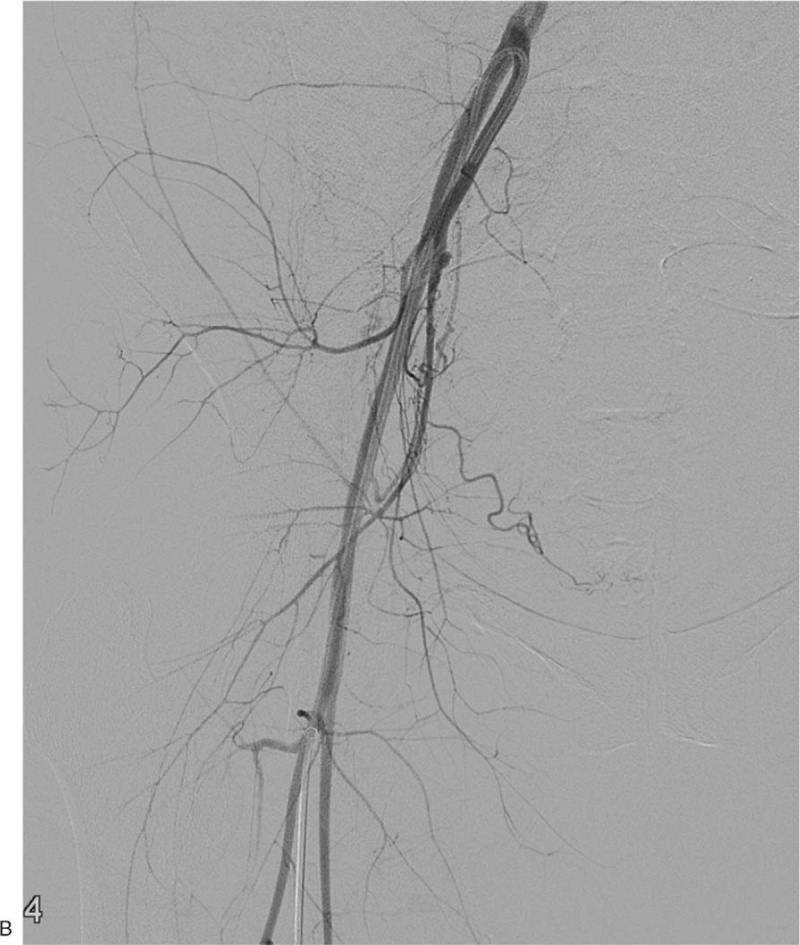
A 34-year-old woman (gravida 2, para 2) transferred to our hospital for persistent vaginal bleeding after cesarean section. (A and B) Both uterine arteries are not hypertrophied despite recent postpartum state, and there is no evidence of vascular injuries such as active bleeding, pseudoaneurysm, or transection of vessel on the angiographies of both internal iliac arteries.

**Figure 2:**
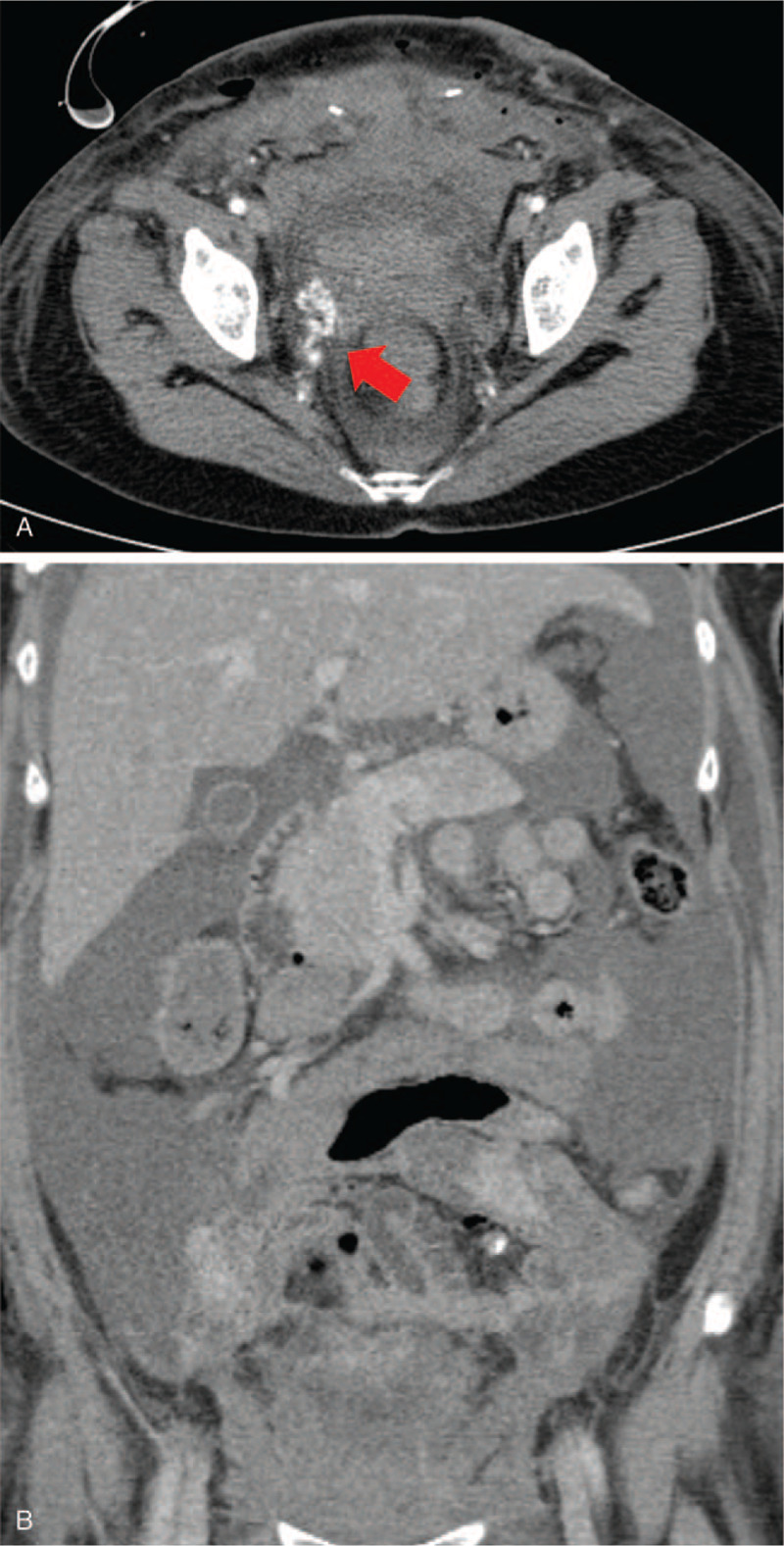
A 34-year-old woman (gravida 2, para 2) transferred to our hospital for persistent vaginal bleeding after cesarean section. The computed tomography (CT) scan 2 days after hysterectomy shows abnormal tortuous hypertrophied vascular structure at right lateral aspect of the pelvic cavity (red arrow) on axial image (A) and a large amount of hemoperitoneum on coronal image (B).

**Figure 3:**
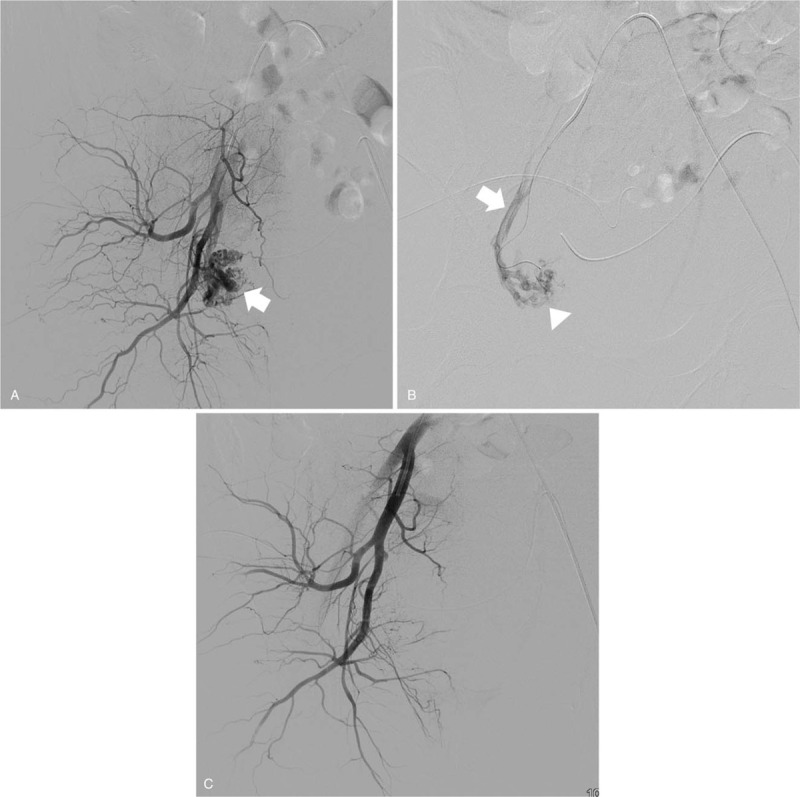
A 34-year-old woman (gravida 2, para 2) transferred to our hospital for persistent vaginal bleeding after cesarean section. (A) The right internal angiography shows a remnant right uterine artery (UA) and a complex tangle of vessels supplied by an enlarged feeding artery (arrow) at its distal portion. (B) Superselective uterine artery angiography shows early venous drainage into hypertrophied veins (white arrow), and stasis of contrast medium in the abnormal vascular structure (white arrow head), which indicates UVM. (C) A post-embolization angiogram reveals successful hemostasis with disappearance of the UVM. UVM = uterine arteriovenous malformation.

## 3 Discussion

Uterine AVM is a relatively rare disorder and the documented information of it is still sparse.^[1,2]^ Acquired UVM is the predominant type. It consists of multiple small arteriovenous fistulas between intramural arterial branches and the myometrial venous plexus, tends to have single or bilateral uterine artery feeders without an extrauterine arterial supply, and does not have a characteristic nidus.^[1–3]^ Acquired UVM has been reported after therapeutic abortion, uterine surgery, cesarean section, direct uterine trauma, and gestational trophoblastic disease.^[2]^ Acquired UVM has also been reported, although rarely, after hysterectomy, and published cases report symptoms of UVM presenting from 3 months up to 35 years postoperatively.^[6,7]^ To the best of our knowledge, this case is the shortest time interval from procedure to onset of symptoms. In our patient who underwent hysterectomy to control of intractable postpartum bleeding, there was no evidence of a UVM prior to the hysterectomy, nor was there any suggestion of it at the time of surgery or on pathologic examination of postoperative specimens. It was therefore concluded that the pathology occurred as a result of the hysterectomy.

Hysterectomy is one of the most common procedures in gynecological practice and is the best treatment option for intractable postpartum bleeding accompanied by hemodynamic instability or coagulopathy.^[8,9]^ Although hysterectomy is the most invasive option and the last resort, bleeding may recur or be persistent after the procedure despite advanced surgical techniques. Possible reasons for this include improperly ligated vessels, effects of hemoperitoneum, use of the Trendelenburg position, bleeding from an anastomotic site or cut surface, low intraoperative blood pressure, subacute infection, bleeding disorders, and rarely, UVM such as that presented in our case.^[9]^ Therefore, it is expected that a careful and informed approach, based on awareness of the various possible causes of intractable postpartum bleeding even after hysterectomy, could increase the success rate of treatment.

The diagnosis of UVM is often difficult and requires a high index of suspicion. Imaging methods such as CT scan and MRI may be used to establish the diagnosis. These imaging techniques will also help to determine the size, extent, and vascularity of the lesion as well as the involvement of adjacent organs. CT scan is usually used as the initial diagnostic modality for the detection of a bleeding focus after surgery.^[1]^ Characteristic CT scan findings include the presence of a soft-tissue dense mass with an enhancement pattern resembling adjacent vessels.^[2]^ It may help the intervention radiologist to plan for the procedure, and is therefore likely to decrease the number of angiograms required to localize the bleeding site and the radiation dose received by the patient. A recent study reported that CT scan revealed active bleeding more frequently than angiography.^[2]^ Our case represents a complementary role of CT scan and angiography in the management of postoperative bleeding of a patient who underwent hysterectomy. Here the CT scan after surgery revealed a tortuous hypertrophied vascular structure suggesting UVM at the right lateral aspect of the pelvic cavity. Angiography should be offered for consideration as a possible therapeutic option following diagnostic confirmation. The typical finding of UVM by angiography is a high arterial flow with early venous filling. A single direct fistulous communication to the venous structures may be identified. This finding is more common in acquired UVM than in congenital UVM,^[1]^ where contrast filling of a vascular plexus or nidus is more commonly seen.

A wide spectrum of treatment plans have been proposed for UVM including observation, use of oral medications, endovascular embolization, laparoscopic coagulation, and surgical ligation of uterine arteries.^[3]^ The management of choice is decided according to hemodynamic stability or the need to control an acute, heavy hemorrhage. In our case, the patient experienced recurrence of bleeding 2 days after hysterectomy. Traditionally, postoperative recurrence of bleeding is treated with repeat surgery.^[2]^ However, the disadvantages include the need for general anesthesia for a hemodynamically unstable patient, and secondary surgical complications that include infection, bleeding, and ureteral injury that may occur in an emergency operation. In the last decade, an increasing number of published case reports have chosen to treat UVM by TAE, and the clinical success rate is greater than 90%, with a 4% complication rate in retrospective review articles.^[2]^ Compared with surgical procedure, TAE is a safe and effective treatment option which includes low procedure-related complication rates, avoidance of surgical risks, and shorter hospitalization.^[3]^ The standard agent used for treatment of UVM is NBCA, an agent that enables controlled and permanent obliteration of the UVM.^[3]^ NBCA is a liquid ester that polymerizes rapidly in the presence of ionic substances like blood or saline and has been effectively used to treat gastrointestinal tract bleeding, intrapelvic bleeding, and tumors.^[8,9]^ NBCA is mixed with iodized oil at a ratio ranging from 1:1 to 1:10, depending on the arteriovenous transit time and rate of polymerization desired. An appropriate mixture can occlude the feeding and draining vessels after a single injection.^[3]^ Other agents such as absorbable gelatin sponges a polyvinyl alcohol have also have been used successfully for embolization, especially when the target artery is very distal and further catheter advancement is difficult or impossible.^[1]^ We used a 1:4 mixture NBCA to emblaze the uterine artery and complete occlusion of the artery was confirmed on completion of angiography and there was no evidence of procedure related complication or recurrent bleeding after the TAE.

## 4 Conclusion

In conclusion, UVM is a rare but potentially serious cause of abnormal intraperitoneal bleeding and should be considered as a rare cause of post-hysterectomy bleeding; therefore, a high index of clinical suspicion is required, based on awareness of the various possible cause of UVM, especially when the patient has refractory postpartum hemorrhage. In this situation, TAE is a safe and effective treatment of choice in patients with massive bleeding caused by acquired UVM.

## Author contributions

Conceptualization: Chang Hoon Oh, Yook Kim, Bum Sang Cho

Resources: Yook Kim

Data curation: Chang Hoon Oh, Yook Kim

Formal analysis: Chang Hoon Oh, Yook Kim, Kyung Sik Yi

Supervision: Yook Kim, Bum Sang Cho, Kyung Sik Yi

Writing - original draft: Chang Hoon Oh, Yook Kim

Writing - review & editing: Chang Hoon Oh, Yook Kim

Yook Kim orcid: 0000-0003-2162-419X.

## Abbreviations

Abbreviations: CT = computed tomography, NBCA = n-butyl cyanoacrylate, TAE = transcatheter arterial embolization, UVM = uterine arteriovenous malformation.
